# STOP-Bang Modification for Screening Treatment-Relevant Obstructive Sleep Apnea in Cluster Headache: A Retrospective Cohort Study

**DOI:** 10.3390/jcm15155952

**Published:** 2026-07-30

**Authors:** Mi-Kyoung Kang, Yooha Hong, Hee-Jin Im, Jinseob Kim, Soo-Jin Cho

**Affiliations:** 1 Department of Neurology, Dongtan Sacred Heart Hospital, Hallym University College of Medicine, Hwaseong-si 18450, Republic of Korea; alroddlbebe@gmail.com (M.-K.K.); dbgk486@naver.com (Y.H.); coolere@naver.com (H.-J.I.); 2 Zarathu Co., Ltd., Seoul 05854, Republic of Korea

**Keywords:** age of onset, cluster headache, diagnosis, obstructive sleep apnea, polysomnography, smoking

## Abstract

**Background**: Cluster headache (CH) and obstructive sleep apnea (OSA) are both associated with elevated cardiovascular risk and unhealthy lifestyle factors, including smoking and alcohol use. This study investigated whether the conventional STOP-Bang questionnaire or a modified screening approach incorporating CH-specific clinical features is more useful for identifying treatment-relevant OSA. **Methods**: We retrospectively analyzed 52 patients with CH who underwent polysomnography (PSG) between January 2020 and December 2025. An apnea–hypopnea index (AHI) of at least 10 events/hour was defined as treatment-relevant OSA. We assessed whether incorporating age at CH onset and the circadian and/or seasonal rhythmicity of CH attacks into the STOP-Bang questionnaire improved screening performance. **Results**: Of 52 patients, 41 (78.8%) were diagnosed with OSA (AHI ≥ 5) and 25 (48.1%) had treatment-relevant OSA. Patients with treatment-relevant OSA were predominantly male and had a higher median age, body mass index, and age at CH onset. A modified model incorporating CH onset at or after age 30 showed modest improvement than conventional STOP-Bang. At a cut-off point of ≥3, the modified model demonstrated a numerically higher area under the curve (0.804 vs. 0.767) and greater sensitivity (84.0% vs. 76.0%; DeLong *p* = 0.125), with low specificity (48.1% vs. 55.6%). At a cut-off point of ≥4, the specificity increased to 77.8%. The addition of other clinical characteristics of CH did not further improve the performance. **Conclusions**: Incorporating CH onset age into the STOP-Bang modestly improved screening for treatment-relevant OSA in CH patients, representing a practical screening refinement warranting external validation.

## 1. Introduction

Cluster headache (CH) is a primary headache disorder defined by the 3rd edition of the International Classification of Headache Disorders (ICHD-3) as attacks of severe, strictly unilateral pain lasting 15–180 min, accompanied by ipsilateral autonomic symptoms and/or restlessness [1], and characterized by striking circadian and circannual periodicity, excruciating pain intensity, and substantial functional impairment. It is widely regarded as one of the most disabling headache syndromes, with profound impact on quality of life and daily functioning [2]. CH typically has an onset in the third to fourth decade of life and shows a male predominance, with reported male-to-female ratio ranging from approximately 2:1 to 4:1 across population-based studies [3,4].

Obstructive sleep apnea (OSA) has been increasingly recognized as a common comorbidity in patients with CH. Previous studies have reported OSA prevalence rates ranging from approximately 58% to 80% in CH cohorts undergoing polysomnography, suggesting a potential pathophysiological link beyond shared risk factors [5,6,7,8]. Both conditions exhibit nocturnal predominance, are more common in men, and are associated with unhealthy lifestyle factors, such as smoking and alcohol consumption [7,9,10]. Untreated OSA is associated with increased risks of cerebrovascular disease, cardiovascular morbidity [11], and mortality [12]. Importantly, patients with CH are reported to be at an increased risk of hypertension and coronary heart disease [13]. They also have high smoking rates and are at risk of potential medication overuse, which may further exacerbate the consequences of unrecognized OSA. Therefore, failure to identify and manage OSA in this population may adversely affect both headache outcomes and systemic comorbidities.

Although several case reports have described improvement of CH symptoms following treatment of OSA using positive airway pressure (PAP), systematic and controlled evidence remains limited [14,15,16]. To evaluate the clinical necessity and therapeutic implications of OSA in patients with CH, it is important to identify treatment-relevant OSA rather than considering all cases of OSA equally. While an AHI of ≥15 events/hour is generally used to define moderate OSA, treatment decisions often incorporate symptom burden and comorbidities in addition to AHI. Therefore, rather than focusing solely on conventional OSA severity categories, we adopted an AHI ≥ 10 events/hour as a pragmatic definition of treatment-relevant OSA, which may better reflect real-world clinical practice in this population. Patients with CH frequently experience nocturnal attacks, sleep disruption, insomnia, or excessive daytime sleepiness, which may justify PAP treatment even at AHI levels below 15. This approach is consistent with the American Thoracic Society Research Statement, which recognizes that symptomatic patients with mild OSA may derive meaningful benefits from treatment, as well as longitudinal studies demonstrating progressively increasing cardiovascular risk across the mild-to-moderate OSA range. Therefore, an AHI ≥ 10 was considered more appropriate for the treatment-oriented screening objective of the present study than conventional OSA thresholds [17,18]. Furthermore, evidence from both longitudinal and cross-sectional studies suggests that cardiovascular risk may increase even at AHI levels below the conventional moderate OSA threshold (AHI < 15) [19,20]. Although these findings were not derived from patients with CH and therefore should not be directly generalized to this population, they provide additional rationale for exploring an AHI ≥ 10 threshold in a treatment-oriented screening strategy for patients with CH.

OSA itself is frequently underdiagnosed and undertreated, with substantial delays in diagnosis [21]. The STOP-Bang questionnaire is a widely used screening tool for OSA, known for its high sensitivity in general and preoperative populations [22,23]. However, its diagnostic performance may be reduced in specific populations, particularly in conditions with high OSA prevalence such as atrial fibrillation, or in non-obese individuals [24,25]. Given that CH typically affects individuals in their third to fourth decades of life and is not strongly associated with severe obesity [26], the applicability and performance of the STOP-Bang questionnaire in this population warrant further evaluation.

In this context, the present study aimed to evaluate the diagnostic utility of the STOP-Bang questionnaire in patients with CH and to explore whether incorporating disease-specific clinical variables can refine its performance as a screening tool for treatment-relevant OSA in this population. We hypothesized that a CH-specific modification of STOP-Bang would improve screening performance for treatment-relevant OSA.

## 2. Methods

### 2.1. Study Design and Participants

This retrospective observational study included consecutive patients with CH who underwent PSG based on clinical suspicion of OSA or the treating physician’s clinical judgment at the Headache Clinic of the Department of Neurology, Hallym University Dongtan Sacred Heart Hospital (Hwaseong, Republic of Korea), between January 2020 and December 2025. PSG was performed when clinically indicated because of suspected sleep-disordered breathing or at the discretion of the treating physician based on the overall clinical assessment.

The study protocol was approved by the Institutional Review Board of Hallym University Dongtan Sacred Heart Hospital (IRB No. 2022-12-003-001. Initial approval date: 25 January 2023, final amendment date: 12 March 2026). Written informed consent was obtained from all participants included in the prospective Korean Cluster Headache Registry (KCHR IRB No 2016-09-396. Initial approval date: 21 September 2016, final amendment date:v19 June 2025) [27]. For patients who were not enrolled in the KCHR, only de-identified retrospective data were collected and analyzed, and the requirement for informed consent was waived by the Institutional Review Board. As PSG was performed only in patients with clinical indications, including suspected OSA or the treating physician’s clinical judgment, this study is subject to partial verification bias, and diagnostic estimates apply to this enriched subgroup rather than an unselected CH population.

CH was diagnosed by board-certified neurologists according to the diagnostic criteria of the ICHD-3 [1]. All patients underwent neuroimaging (MRI or CT) to exclude secondary causes of headache; fundoscopic examination, ophthalmological referral, and lumbar puncture were obtained selectively when secondary causes such as idiopathic intracranial hypertension were clinically suspected. As this was a retrospective study based on data extracted from the KCHR and medical records, patients were not followed prospectively for a defined follow-up period. Inclusion criteria were age ≥ 19 years (the legal age of adulthood in Korea and the KCHR enrollment criterion), diagnosis of episodic or chronic CH according to ICHD-3, and completion of PSG evaluation. Exclusion criteria were incomplete PSG recording or missing STOP-Bang item data required for score calculation.

### 2.2. Clinical Assessment

Clinical and demographic data were collected using structured questionnaires and medical record review. Variables included age, sex, body mass index (BMI), smoking history, alcohol consumption, disease duration, age at CH onset, headache intensity assessed using a visual analog scale (VAS), attack frequency, and circadian or seasonal rhythmicity.

The STOP-Bang questionnaire was used as the conventional screening tool for OSA [22]. The questionnaire includes eight items: snoring, tiredness, observed apnea, hypertension, BMI >35 kg/m^2^, age >50 years, neck circumference, and male sex. Total STOP-Bang scores were calculated as the sum of positive items.

We constructed modified screening models by incorporating CH-specific clinical features, including age, at CH onset and the circadian and/or seasonal rhythmicity of CH attacks, into the conventional STOP-Bang questionnaire and evaluated their performance for screening treatment-relevant OSA.

CH onset age ≥ 30 years (C30) was evaluated as a candidate screening item. C30 was added as one unweighted point to the 8-item STOP-Bang score (9 items total), with the same cutoff thresholds (e.g., ≥3, ≥4) applied directly to the combined score. The 30-year threshold was informed by epidemiological data showing that CH onset clusters around the third decade (mean ~30 years) [3,4], together with our own finding that age at CH onset was significantly higher in patients with treatment-relevant OSA. Circadian rhythmicity, nocturnal rhythmicity (22:00–5:59), morning rhythmicity (6:00–9:59), and seasonal rhythmicity were evaluated separately. Nocturnal and morning rhythmicity were additionally analyzed as subtypes of circadian rhythmicity. Patients were considered to have circadian and/or seasonal rhythmicity if circadian rhythmicity, seasonal rhythmicity, or both were present.

### 2.3. Polysomnography Assessment

All participants underwent attended overnight laboratory PSG using a standard level 1 sleep study system (Embla^®^; Natus Medical Inc., Pleasanton, CA, USA). Recorded parameters included electroencephalography, electrooculography, electromyography, electrocardiography, airflow, respiratory effort, pulse oximetry, body position, and limb movements. PSG recordings were scored by board-certified sleep specialists according to the American Academy of Sleep Medicine Manual for the Scoring of Sleep and Associated Events (version v2.6, 2020) apnea was defined as a ≥90% reduction in airflow from baseline lasting ≥10 s. Hypopnea was defined as a ≥30% reduction in airflow from baseline lasting ≥10 s accompanied by a ≥3% oxygen desaturation or an arousal [Recommended rule 1A] [28]. The AHI was calculated as the number of apnea and hypopnea events per hour of sleep. OSA was defined as AHI ≥ 5 events/hour. To avoid repeated measurements from the same individual, only the first PSG study was included in the analysis for patients who underwent PSG on multiple occasions. STOP-Bang scores were reconstructed retrospectively from structured questionnaires and medical records, whereas PSG studies were scored as part of routine clinical care; the index test (STOP-Bang) and the reference standard (PSG) were therefore obtained and interpreted independently. STOP-Bang items were extracted without reference to the PSG-derived AHI.

### 2.4. Statistical Analysis

No formal sample size calculation was performed; the study included all consecutive patients who underwent PSG during the study period. Continuous variables were summarized as median (interquartile range, IQR), and categorical variables were summarized as number (percentage). Between-group comparisons were performed using the Wilcoxon rank-sum test for continuous variables and Fisher’s exact test for categorical variables. Diagnostic performance was assessed using PSG-confirmed OSA as the reference standard, primarily defined as an AHI ≥ 10 events/hour, with AHI ≥ 5 events/hour used as a sensitivity outcome.

Receiver operating characteristic curves were constructed for the original STOP-Bang score and modified screening scores. The area under the curve (AUC) and 95% confidence intervals were estimated, and paired AUC comparisons were performed using the DeLong test. The incremental value of candidate clinical features over the STOP-Bang score was evaluated using nested logistic regression models compared by likelihood-ratio tests, which served as the formal test of added value. Continuous net reclassification improvement was calculated only as an exploratory measure; because it is unstable in small samples, it was not used as primary evidence of model superiority.

Because no external validation cohort was available, the discrimination of the screening scores was internally checked using bootstrap resampling with optimism correction (Harrell’s enhanced bootstrap, 2000 resamples). For each resample a logistic model was refit and its AUC reestimated on the resample and on the original cohort; the mean difference (optimism) was subtracted from the apparent AUC. Conditional on the pre-specified C30 rule, optimism was negligible for the primary outcome (optimism-corrected AUC 0.77 for STOP-Bang and 0.80 for STOP-Bang + C30 at AHI ≥ 10 events/h), consistent with the use of pre-specified unweighted point scores that estimate no weights from the data; a stricter model that re-estimated separate weights yielded an optimism-corrected AUC of 0.81. Because C30 was identified in this same cohort by screening candidate CH items against STOP-Bang, the candidate selection was additionally repeated within each resample, and the selection-aware optimism was likewise negligible (optimism-corrected AUC 0.80). This internal validation does not replace external validation: as C30 was derived in this single cohort, the modified score should be regarded as exploratory and hypothesis-generating, and external validation in an independent CH population is required. All PSG recordings yielded complete AHI values, and all STOP-Bang items were fully ascertainable from medical records; no indeterminate results occurred. All analyses were performed using R 4.5.2 (R Foundation for Statistical Computing, Vienna, Austria).

## 3. Results

### 3.1. Patient Characteristics According to Treatment-Relevant OSA

**Figure 1:**
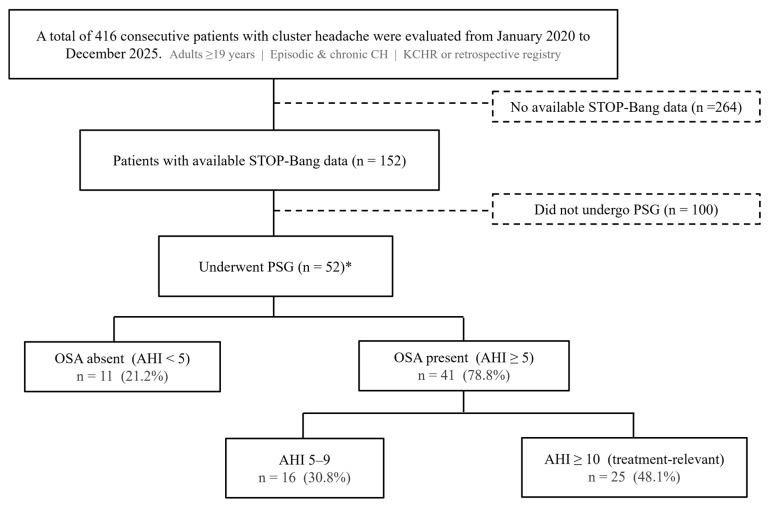
Flow diagram of participant enrollment and classification. * Only the first PSG was included in patients with multiple recordings. PSG was performed during the active bout (n = 28) or remission (n = 24). Indications for PSG included suspected sleep-disordered breathing or the treating physician’s clinical judgment based on the overall clinical assessment. AHI, apnea–hypopnea index; CH, cluster headache; KCHR, Korean Cluster Headache Registry; OSA, obstructive sleep apnea; PSG, polysomnography.

Among 416 consecutive patients with CH, 52 underwent PSG based on clinical suspicion of OSA or the treating physician’s clinical judgment, and those who underwent PSG were predominantly male compared with those who did not undergo PSG (90.4% vs. 73.9%, *p* = 0.011). There was no significant difference in age between the two groups (39.2 ± 9.3 vs. 39.9 ± 11.8 years, *p* = 0.68). Male sex is a recognized clinical risk factor for OSA, which may partly explain this difference in referral for PSG. Among a total of 52 patients with CH included in this study, 41 (78.8%) were diagnosed with OSA (AHI ≥ 5), and 25 (48.1%) were identified as having treatment-relevant OSA (AHI ≥ 10) (Figure 1). Patients with treatment-relevant OSA exhibited a significantly higher median age at CH onset compared to those without (32.00 vs. 21.00 years, *p* = 0.001). Additionally, the STOP-Bang score was significantly higher in the OSA group (median 4.00 vs. 2.00, *p* = 0.001). All patients in the treatment-relevant OSA group were male (100.0%), compared with 81.5% in the non-OSA group, a difference that approached but did not reach statistical significance (*p* = 0.052). BMI, headache severity (VAS), and circadian rhythmicity did not differ between the groups, whereas disease duration (15.00 vs. 10.00 years, *p* = 0.077) and seasonal rhythmicity (70.4% vs. 40.0%, *p* = 0.054) also showed trends toward a difference without reaching statistical significance (Table 1).

### 3.2. Diagnostic Performance of STOP-Bang and Individual Items

The diagnostic utility of the conventional STOP-Bang questionnaire was evaluated for identifying treatment-relevant OSA. At a cutoff score of ≥3, the STOP-Bang demonstrated a sensitivity of 76.0% and a specificity of 55.6%. Analysis of individual items revealed that “Male sex” (G) provided the highest sensitivity (100.0%) but very low specificity (18.5%). In contrast, “BMI ≥ 35” (B) and “Neck circumference” (N) exhibited 100.0% specificity, making them useful for ruling in the diagnosis, though their sensitivities were extremely low (0.0% and 12.0%, respectively, Table 2).

When OSA was defined using the conventional AHI threshold of ≥5, the STOP-Bang at a cutoff of ≥3 yielded a sensitivity of 63.4% and a specificity of 54.5%, suggesting limited diagnostic utility in this CH population (Supplementary Table S1).

### 3.3. Performance of Modified Screening Models

Among the CH-specific clinical features evaluated, C30 showed moderate diagnostic performance for treatment-relevant OSA, with a sensitivity of 64.0% and specificity of 81.5%. In contrast, circadian/seasonal rhythmicity demonstrated high sensitivity (76.0%) but extremely low specificity (3.7%), resulting in poor overall discriminative performance (Table 3). Neither circadian, nocturnal, morning, nor seasonal rhythmicity provided incremental value for identifying treatment-relevant OSA beyond the STOP-Bang questionnaire (Supplementary Table S2 and Figure S1).

**Figure 2:**
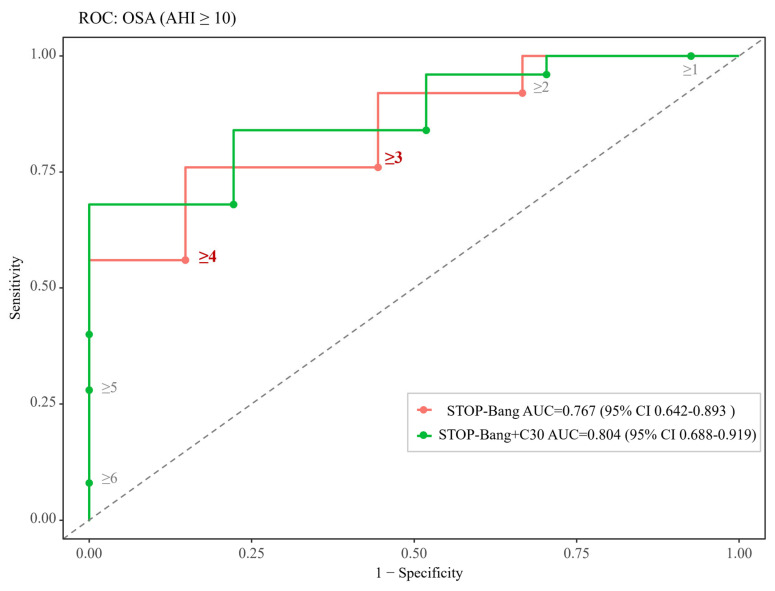
Receiver operating characteristic (ROC) curves of the conventional STOP-Bang questionnaire (salmon) and the modified STOP-Bang + C30 model (green) for identifying treatment-relevant obstructive sleep apnea (apnea–hypopnea index ≥10 events/hour) in patients with cluster headache (CH) (n = 52). The conventional STOP-Bang achieved an area under the curve (AUC) of 0.767 (95% CI, 0.642–0.893) and the STOP-Bang + C30 model an AUC of 0.804 (95% CI, 0.688–0.919); this difference was not statistically significant (DeLong *p* = 0.125), although the incremental value of C30 was supported by likelihood-ratio testing (*p* = 0.011). Labeled points indicate successive STOP-Bang score thresholds (≥1 to ≥6); the cutoffs, ≥3 and ≥4, are highlighted in bold red. The dashed diagonal represents the line of no discrimination. C30, CH onset age ≥30 years; CI, confidence interval.

Based on the finding that CH onset age significantly differed by OSA status, a modified model was developed by incorporating “CH onset age ≥ 30” (C30). When applying a cutoff of ≥3, the STOP-Bang + C30 model demonstrated higher sensitivity of 84.0% (vs. 76.0%) and slightly lower specificity (48.1% vs. 55.6%) (Table 3). The AUC was numerically higher for STOP-Bang + C30 than for STOP-Bang (0.804 vs. 0.767), but this difference was not statistically significant (DeLong *p* = 0.125; Figure 2). In contrast, the incremental value of adding C30 to STOP-Bang was statistically significant by the likelihood-ratio test (*p* = 0.011; adjusted OR 5.58, 95% CI 1.40–22.28), indicating that C30 contributes independent information beyond the STOP-Bang score. After bootstrap optimism correction (2000 resamples), including a selection-aware bootstrap that repeated the candidate selection within each resample, discrimination was essentially unchanged (optimism-corrected AUC 0.80 for STOP-Bang + C30 and 0.77 for STOP-Bang; Supplementary Table S3). Continuous NRI for C30 (0.910, 95% CI 0.433–1.387; *p* < 0.001) was directionally consistent with the likelihood-ratio result, whereas none of the rhythmicity features showed significant positive NRI, with circadian and/or seasonal rhythmicity showing a negative NRI of −1.064 (95% CI −1.517 to −0.610; *p* < 0.001), consistent with its poor discriminative performance (Supplementary Table S2). Given the limited sample size, NRI findings are interpreted only as exploratory and supportive, with the likelihood-ratio test serving as the primary evidence for the incremental value of C30.

Furthermore, increasing the cutoff to ≥4, improved the specificity to 77.8% and increased the positive likelihood ratio to 3.06, suggesting better rule-in performance for treatment-relevant OSA in CH patients (Table 3).

When OSA was defined using AHI ≥ 5, the STOP-Bang + C30 at a cutoff of ≥3 demonstrated a sensitivity of 73.2% and a specificity of 54.5%, suggesting better performance than the STOP-bang itself (Supplementary Table S4).

### 3.4. Subgroup Analysis by Polysomnography Timing

PSG was performed during an active cluster bout in 28 patients and during a remission period in 24 patients. The two subgroups were comparable with respect to AHI (7.8 [5.1–17.6] vs. 10.6 [5.2–23.6] events/hour, *p* = 0.693), STOP-Bang score (3 [2,3,4] vs. 3 [2,3,4], *p* = 0.620), and treatment-relevant OSA prevalence (12/28, 42.9% vs. 13/24, 54.2%, *p* = 0.578) (Supplementary Table S5).

Diagnostic performance was then assessed according to PSG timing (Table 4). In patients who underwent PSG during an active cluster bout (n = 28), both the STOP-Bang (AUC = 0.820) and the STOP-Bang + C30 (AUC = 0.823) showed high diagnostic accuracy. For patients evaluated during a remission period (n = 24), the STOP-Bang + C30 achieved a numerically higher AUC (0.755) compared to the conventional STOP-Bang (0.685). At a cutoff of ≥3, adding C30 numerically increased sensitivity in both subgroups (bout: 83.3% vs. 75.0%; remission: 84.6% vs. 76.9%) with comparable specificity (bout: 50.0% vs. 62.5%; remission: 45.5% vs. 45.5%).

## 4. Discussion

To our knowledge, this is among the largest PSG-based studies evaluating treatment-relevant OSA screening in patients with CH, and the first to reflect real-world PSG utilization rates by including the entire clinical CH population from which the examined cohort was drawn. Among CH patients referred for PSG, OSA of any severity (AHI ≥ 5) was present in 78.8% and treatment-relevant OSA (AHI ≥ 10) in nearly half (48.1%). Furthermore, we developed a modified screening model, STOP-Bang with CH onset age ≥ 30 years, which incorporates CH-specific feature as an additional item. Although the AUC gain over conventional STOP-Bang was not statistically significant (DeLong *p* = 0.125), C30 showed a statistically significant incremental value by likelihood-ratio test (*p* = 0.011), supporting its contribution as an additional predictor.

While prior PSG studies in CH have included between 12 and 42 patients, this study represents the largest cohort to date (n = 52). Furthermore, by documenting that only 12.5% of a consecutive clinic-based CH population (n = 416) underwent PSG, this study provides real-world context regarding the gap between clinical suspicion and formal sleep evaluation in this population.

The prevalence of OSA in this study was consistent with previously reported rates of 58–80% in PSG-based CH cohorts [6,8,29,30]. Several mechanisms have been proposed in the literature to explain the coexistence of OSA and CH, including intermittent hypoxia and hypothalamic dysfunction; however, causal relationships remain uncertain, and this cross-sectional study was not designed to test such mechanisms [31,32]. Both conditions have separately been linked to hypothalamic involvement, though whether this reflects a shared mechanistic pathway remains speculative and requires dedicated mechanistic studies. Despite the relatively low rate of PSG evaluation, the high prevalence of OSA among tested patients underscores the potential clinical value of OSA screening in individuals with CH, particularly those with clinical features suggestive of sleep-disordered breathing.

A key finding of our study was that patients with treatment-relevant OSA had a significantly higher median CH onset age compared to those without (32.00 vs. 21.00 years). This observation formed the rationale for incorporating “CH onset age ≥ 30 years” (C30) into a modified screening model. One possible explanation is that later CH onset reflects a phenotype with greater metabolic burden, weight gain, or upper airway susceptibility-established OSA risk factors; however, this remains speculative, as our study did not directly assess these mechanisms. Given that the discriminative cutoff identified in our cohort (CH onset age ≥ 30 years) is substantially younger than the age at which OSA risk typically increases in the general population [12], further mechanistic studies are needed to clarify this association.

Circadian and seasonal rhythmicity, hallmark features of CH, did not provide incremental diagnostic value for OSA identification beyond the STOP-Bang. Nocturnal and morning headache patterns have been hypothesized in the literature to reflect OSA-related arousal mechanisms [33]; our data, however, did not support the use of these features as screening markers and cannot confirm or refute this proposed mechanism. This may reflect the fact that high attack frequency and circadian or seasonal rhythmicity are common features of CH, thereby limiting their discriminatory value for identifying coexisting OSA.

The conventional STOP-Bang questionnaire demonstrated modest screening and discriminating performance in this CH population. This is partly attributable to the demographic characteristics of CH patients, who are predominantly male and non-obese. The “Male sex” item, while providing near-zero specificity (18.5%) and while BMI ≥ 35 and neck circumference showed high specificity but negligible sensitivity. In the present study, the STOP-Bang questionnaire at a cutoff of ≥3 achieved an AUC of 0.767 and a sensitivity of 76.0% for treatment-relevant OSA. When OSA was defined as AHI ≥ 5, the sensitivity at the same cutoff was 63.4%. Where the STOP-Bang typically demonstrates AUC values of approximately 0.70–0.80 and sensitivity ranging from 73% to 94% in general and sleep-clinic populations [22,23], the sensitivity of STOP-Bang was somewhat lower in this population. These findings suggest that while conventional OSA risk factors retain relevance in CH, disease-specific variables may further refine screening performance.

Consistent with our findings, Lund et al. reported that neither AHI nor OSA prevalence differed significantly between cluster bout (n = 32) and remission (n = 23), whether compared across patients or within the same patients studied in both phases (n = 14) [34]. In our cohort as well, AHI, STOP-Bang scores, and OSA prevalence were comparable between patients studied in the two phases, supporting screening irrespective of the timing of evaluation.

When the modified model was applied by PSG timing, the STOP-Bang + C30 retained diagnostic performance during remission without a substantial decline relative to the active bout. Because age at CH onset does not change over the course of the disease, it provides a stable screening feature that is largely unaffected by the disease phase at the time of testing—a practical advantage, given that PSG in CH may be performed during either an active bout or remission. The lower performance of the conventional STOP-Bang during remission may not necessarily reflect a true decline in accuracy, as the bout and remission groups comprised different patients, and differences in patient composition may have contributed. Given the small subgroups (n = 28 and n = 24) and wide, overlapping CI (Table 4), these differences should be regarded as descriptive rather than confirmatory, and the findings as hypothesis-generating.

The present findings have several practical clinical implications. Although an AHI ≥ 15 is conventionally used to define moderate OSA severity, the present study focused on treatment-relevant OSA (AHI ≥ 10), reflecting a pragmatic clinical approach in which treatment decisions are guided by both AHI and symptom burden rather than severity classification alone. Accordingly, the modified screening model is intended to identify patients who may benefit from further sleep evaluation and consideration of PAP therapy, rather than to redefine conventional OSA severity. Given the high prevalence of clinically significant OSA among CH patients referred for PSG, clinicians should maintain awareness of coexisting sleep-disordered breathing in patients with CH, as untreated OSA is associated with adverse cardiovascular outcomes, suggesting that its identification in CH patients has implications beyond headache management alone. At a cutoff of ≥3, the modified model demonstrated high sensitivity in this cohort and may have potential utility as an initial screening approach. These thresholds should be applied with awareness of the small derivation sample, and external validation is needed before widespread adoption. Notable strengths include the use of attended overnight PSG for OSA diagnosis, structured item-level STOP-Bang scoring, and a primary focus on treatment-relevant OSA (AHI ≥ 10) rather than the conventional AHI ≥ 5 threshold—an approach that better reflects clinical decision-making and has not been consistently adopted in prior CH-OSA studies.

Several limitations should also be acknowledged. First, as is inherent to any PSG-referral-based diagnostic accuracy study, PSG was performed only in patients with clinical suspicion of OSA rather than in all consecutive CH patients, introducing partial verification bias. Second, the sample size was relatively small in subgroup analyses according to PSG timing. Third, because the C30 threshold was derived from the same cohort in which its performance was assessed, external validation in an independent cohort is needed. C30 was identified in this single cohort; although bootstrap internal validation (including a selection-aware procedure) showed negligible optimism, the modified score should be regarded as exploratory and requires external validation. Fourth, the AUC gain over conventional STOP-Bang was not statistically significant, although the incremental value of C30 was supported by likelihood-ratio testing; calibration and decision-analytic evaluation were not performed, and the practical clinical benefit of C30 should therefore be regarded as preliminary pending such evidence and external validation. Fifth, the AHI ≥ 10 threshold used to define treatment-relevant OSA does not correspond to a standard international severity boundary, and replication using AHI ≥ 15 is warranted in future larger cohorts. Finally, longitudinal outcomes following OSA diagnosis, including PAP adherence and headache-related outcomes, were not evaluated. Therefore, whether identification and treatment of clinically significant OSA can improve clinical outcomes in patients with CH remains to be determined in future prospective studies.

## 5. Conclusions

Clinically significant OSA was identified in nearly half of patients with CH who underwent PSG. The conventional STOP-Bang questionnaire demonstrated modest screening performance, while incorporation of CH onset age showed additional improvement, even during remission periods. These findings provide preliminary evidence that incorporating CH onset age into STOP-Bang may improve the identification of treatment-relevant OSA among patients with CH. Prospective multicenter validation is required before clinical implementation.

## Figures and Tables

**Table 1:** Comparison of cluster headache patients based on the presence of treatment-relevant obstructive sleep apnea (apnea–hypopnea index of polysomnography ≥10).

Characteristics	Treatment-Relevant Obstructive Sleep Apnea (AHI ≥ 10)		
	Absent (N = 27)	Present (N = 25)	*p*-Value
Age (years)	37.00 [31.50, 42.00]	41.00 [34.00, 45.00]	0.119
Male sex	22 (81.5)	25 (100.0)	0.052
BMI (kg/m^2^)	25.20 [22.95, 27.35]	26.00 [24.50, 28.70]	0.426
Onset Age	21.00 [16.00, 27.50]	32.00 [24.00, 38.00]	0.001
Disease duration (years)	15.00 [5.50, 21.00]	10.00 [6.00, 13.00]	0.077
CH subtype			
Chronic CH	3 (11.1)	3 (12.0)	1.000
First bout	1 (3.7)	1 (4.0)	1.000
Lifetime cluster bouts	10.00 [4.00, 13.50]	7.00 [3.00, 11.00]	0.147
Attack side			
Left	19 (70.4)	14 (56.0)	0.567
Right	6 (22.2)	9 (36.0)	
Alternating/both	2 (7.4)	2 (8.0)	
Maximal duration of cluster bout (day)	40.00 [30.00, 51.00]	66.00 [39.00, 115.00]	0.073
VAS score (headache severity)	9.00 [8.00, 10.00]	9.00 [7.00, 10.00]	0.328
Duration of CH attack (min)	90.00 [60.00, 120.00]	60.00 [40.00, 120.00]	0.155
Frequency of CH attack (/day)	1.00 [1.00, 1.75]	1.00 [1.00, 1.50]	0.793
Circadian rhythmicity	18 (66.7)	17 (68.0)	1.000
Nocturnal attacks	8 (33.3)	9 (39.1)	0.847
Morning attack	6 (25.0)	3 (13.0)	0.469
Daytime attack	8 (32.0)	7 (30.4)	1.000
Seasonal rhythmicity	19 (70.4)	10 (40.0)	0.054
Hypertension	2 (7.4)	7 (28.0)	0.071
Diabetes	2 (7.4)	0 (0.0)	0.491
Hyperlipidemia	6 (22.2)	10 (40.0)	0.277
Smoking status			
Never smoker	13 (48.1)	9 (36.0)	0.109
Current smoker	11 (40.7)	16 (64.0)	
Previous smoker	3 (11.1)	0 (0.0)	
Regular alcohol drinking	13 (56.5)	15 (62.5)	0.904
Comorbid migraine	11 (42.3)	6 (24.0)	0.276
ESS score	5.00 [3.50, 9.00]	9.00 [4.00, 12.00]	0.042
HIT-6 score	69.00 [64.00, 72.00]	69.00 [61.00, 73.50]	0.892
PHQ-9	6.00 [3.50, 8.50]	5.00 [3.00, 9.00]	0.854
GAD-7	4.00 [1.50, 8.50]	4.00 [1.00, 7.00]	0.720
STOP-Bang score	2.00 [1.00, 3.00]	4.00 [3.00, 5.00]	0.001
PSG during cluster bout	16 (59.3)	12 (48.0)	0.592
Lowest oxygen saturation (%)	91.00 [89.00, 92.50]	84.00 [81.00, 86.00]	<0.001
Mean oxygen saturation (%)	96.10 [95.35, 96.75]	94.90 [94.00, 95.70]	0.001

Median (IQR) or N (%). *p* by Wilcoxon rank-sum or Fisher’s exact test. OSA, obstructive sleep apnea; BMI, body mass index; CH, cluster headache; ESS, Epworth Sleepiness Scale; HIT-6, Headache Impact Test-6; PHQ-9, Patient Health Questionnaire-9; GAD-7, Generalized Anxiety Disorder 7-Item Scale; VAS, Visual Analog Scale; PSG, polysomnography. STOP-bang score: snoring, tiredness, observed apnea, high blood pressure, BMI, age, neck circumference, and male gender.

**Table 2:** STOP-Bang items and cutoffs—diagnostic performance for treatment-relevant obstructive sleep apnea (apnea–hypopnea index ≥ 10).

Cutoff	Sensitivity	Specificity	PPV	NPV	LR+	LR−
STOP-Bang ≥ 3	76.0 (56.6–88.5)	55.6 (37.3–72.4)	61.3 (43.8–76.3)	71.4 (50.0–86.2)	1.71	0.43
STOP-Bang ≥ 4	56.0 (37.1–73.3)	85.2 (67.5–94.1)	77.8 (54.8–91.0)	67.6 (50.8–80.9)	3.78	0.52
Snoring (S)	76.0 (56.6–88.5)	48.1 (30.7–66.0)	57.6 (40.8–72.8)	68.4 (46.0–84.6)	1.47	0.50
Tiredness (T)	72.0 (52.4–85.7)	44.4 (27.6–62.7)	54.5 (38.0–70.2)	63.2 (41.0–80.9)	1.30	0.63
Observed apnea (O)	56.0 (37.1–73.3)	81.5 (63.3–91.8)	73.7 (51.2–88.2)	66.7 (49.6–80.2)	3.02	0.54
High BP (P)	24.0 (11.5–43.4)	96.3 (81.7–99.3)	85.7 (48.7–97.4)	57.8 (43.3–71.0)	6.48	0.79
BMI ≥ 35 (B)	0.0 (0.0–13.3)	100.0 (87.5–100.0)	NA	51.9 (38.7–64.9)	∞ *	1.00
Age ≥ 50 (A)	20.0 (8.9–39.1)	92.6 (76.6–97.9)	71.4 (35.9–91.8)	55.6 (41.2–69.1)	2.70	0.86
Neck circumference (N)	12.0 (4.2–30.0)	100.0 (87.5–100.0)	100.0 (43.8–100.0)	55.1 (41.3–68.1)	∞ *	0.88
Male (G)	100.0 (86.7–100.0)	18.5 (8.2–36.7)	53.2 (39.2–66.7)	100.0 (56.6–100.0)	1.23	0.00

Data is presented as average (95% CI). The 95% CIs were estimated using the Wilson method for binomial proportions. Bang = BMI (body mass index), age, neck circumference, and male gender; NPV = negative predictive value; PPV = positive predictive value; STOP = snoring, tiredness, observed apnea, and high BP (blood pressure), NA: Not available due to the absence of subjects meeting the BMI ≥35 criterion. * LR+ was undefined (denoted ∞) when specificity = 100% (zero false positives); LR− was undefined (denoted ∞) only when both sensitivity and specificity = 100%. Otherwise LR− = (1 − sensitivity)/specificity.

**Table 3:** Modified screening models of STOP-Bang items and cutoffs—diagnostic performance for treatment-relevant obstructive sleep apnea (apnea–hypopnea index of polysomnography ≥ 10).

Cutoff	Sensitivity	Specificity	PPV	NPV	LR+	LR−
CH age ≥ 30 (C30)	64.0 (44.5–79.8)	81.5 (63.3–91.8)	76.2 (54.9–89.4)	71.0 (53.4–83.9)	3.46	0.44
Circadian/Seasonal (R)	76.0 (56.6–88.5)	3.7 (0.7–18.3)	42.2 (29.0–56.7)	14.3 (2.6–51.3)	0.79	6.48
STOP + C30 (≥ 3)	64.0 (44.5–79.8)	77.8 (59.2–89.4)	72.7 (51.8–86.8)	70.0 (52.1–83.3)	2.88	0.46
STOP + R (≥ 3)	68.0 (48.4–82.8)	55.6 (37.3–72.4)	58.6 (40.7–74.5)	65.2 (44.9–81.2)	1.53	0.58
STOP-Bang + C30 (≥ 3)	84.0 (65.3–93.6)	48.1 (30.7–66.0)	60.0 (43.6–74.4)	76.5 (52.7–90.4)	1.62	0.33
STOP-Bang + C30 (≥ 4)	68.0 (48.4–82.8)	77.8 (59.2–89.4)	73.9 (53.5–87.5)	72.4 (54.3–85.3)	3.06	0.41

Data is presented as average (95% CI). The 95% CIs were estimated using the Wilson method for binomial proportions. Bang = BMI (body mass index), age, neck circumference, and male gender; NPV = negative predictive value; PPV = positive predictive value; LR = likelihood-ratio, STOP = snoring, tiredness, observed apnea, and high blood pressure.

**Table 4:** Subgroup analysis by polysomnography timing: Diagnostic performance for treatment-relevant obstructive sleep apnea (apnea–hypopnea index of polysomnography ≥ 10).

	OSA (n)	AUC		STOP-Bang ≥ 3		STOP-Bang + C30 ≥ 3	
		STOP-Bang	STOP-Bang + C30	Sensitivity	Specificity	Sensitivity	Specificity
Cluster bout (n = 28)	12	0.820 (0.652–0.989)	0.823 (0.657–0.989)	75.0 (46.8–91.1)	62.5 (38.6–81.5)	83.3 (55.2–95.3)	50.0 (28.0–72.0)
Remission period (n = 24)	13	0.685 (0.474–0.897)	0.755 (0.570–0.940)	76.9 (49.7–91.8)	45.5 (21.3–72.0)	84.6 (57.8–95.7)	45.5 (21.3–72.0)

OSA, obstructive sleep apnea; AUC, area under the curve.

## Data Availability

The datasets generated and/or analyzed during the current study are available from the corresponding author on reasonable request.

## Supplementary Materials

The following supporting information can be downloaded at https://www.mdpi.com/article/10.3390/jcm15155952/s1, Figure S1. Forest plot of the incremental value of CH candidate items over the STOP-Bang questionnaire for treatment-relevant OSA (AHI ≥ 10). Each point represents the adjusted odds ratio (95% CI) for the candidate item from a logistic regression model also containing the STOP-Bang score (OSA~STOP-Bang + item); the vertical dashed line denotes an odds ratio of 1 (no association). Among all candidate items, only CH onset age ≥30 years (C30; red) showed a significant independent association with OSA and was retained in the final model, whereas none of the rhythmicity features (navy) provided significant incremental value (Supplementary Table S2). OR, odds ratio; CI, confidence interval; CH, cluster headache. Table S1. STOP-Bang items and cutoffs for diagnostic performance for obstructive sleep apnea (Apnea Hypopnea index ≥ 5); Table S2. Incremental value of cluster-headache rhythmicity items over STOP-Bang for obstructive sleep apnea (Apnea Hypopnea index ≥ 10); Table S3. Internal validation of screening-score discrimination—bootstrap optimism correction (N = 52); Table S4. Modified screening models for obstructive sleep apnea (Apnea Hypopnea index ≥ 5); Table S5. Polysomnographic and STOP-Bang findings by timing of polysomnography (cluster bout vs. remission).

## Abbreviations

The following abbreviations are used in this manuscript:

CH	cluster headache
OSA	obstructive sleep apnea
PSG	polysomnography;
AHI	apnea–hypopnea index
STOP-Bang	snoring, tiredness, observed apnea, high blood pressure, body mass index, age, neck circumference, male sex.
AUC	area under the curve;
ROC	receiver operating characteristic
BMI	body mass index
C30	cluster headache onset age ≥30 years
CI	confidence interval
KCHR	Korean Cluster Headache Registry
PAP	positive airway pressure
