# Supplementary material for: STOP-Bang Modification for Screening Treatment-Relevant Obstructive Sleep Apnea in Cluster Headache: A Retrospective Cohort Study
Source: J Clin Med. 2026 Jul 30;15(15):5952. doi: 10.3390/jcm15155952 (PMC13467481; doi:10.3390/jcm15155952)
Supplement: Supplementary file 1 [file jcm-15-05952-s001.zip › Figure S1.pdf]

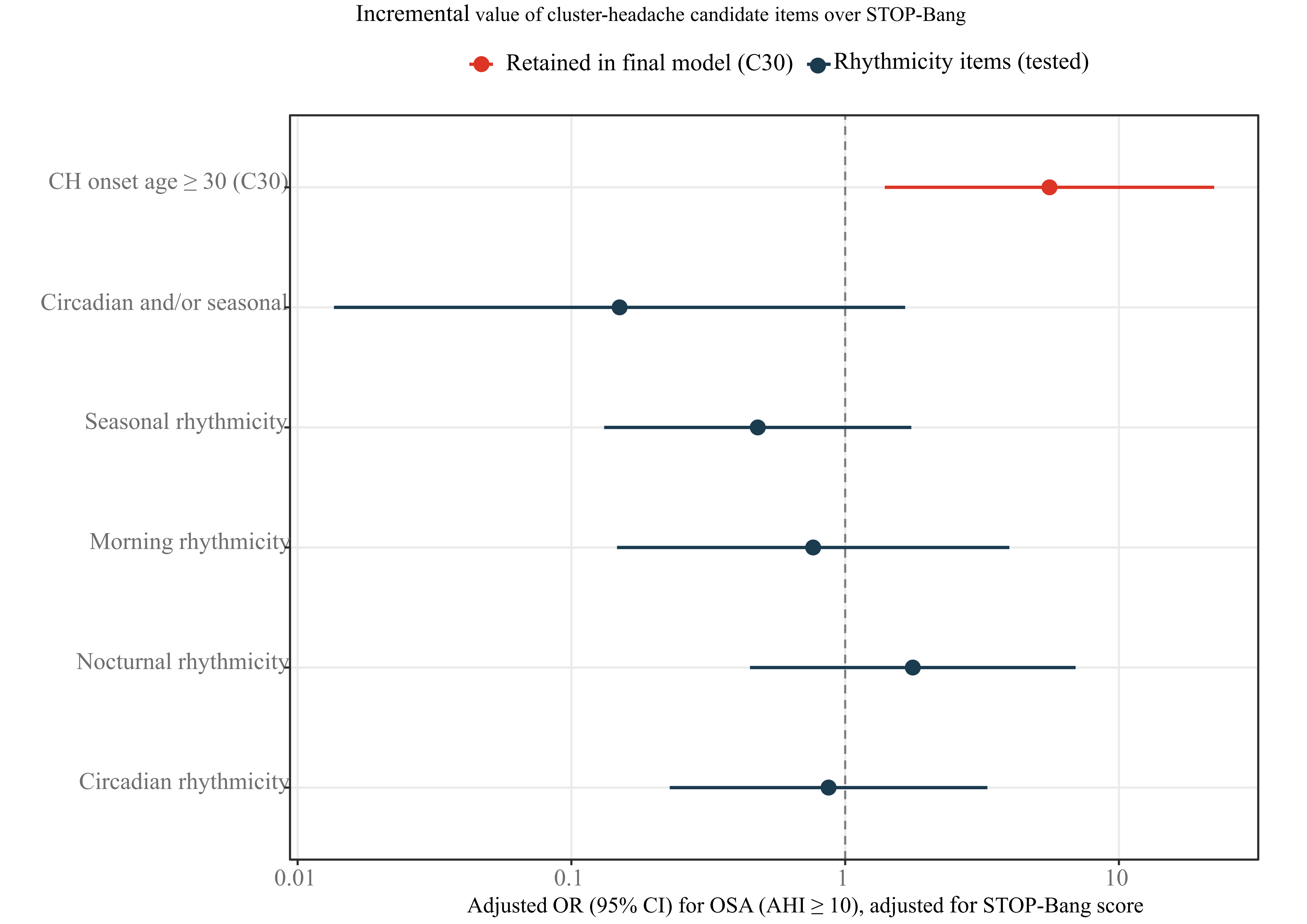

Incremental value of cluster-headache candidate items over STOP-Bang
Retained in final model (C30)
Rhythmicity items (tested)
CH onset age ≥ 30 (C30)
Circadian and/or seasonal
Seasonal rhythmicity
Morning rhythmicity
Nocturnal rhythmicity
Circadian rhythmicity
0.01
0.1
1
10
Adjusted OR (95% CI) for OSA (AHI ≥ 10), adjusted for STOP-Bang score
