# Supplementary material for: STOP-Bang Modification for Screening Treatment-Relevant Obstructive Sleep Apnea in Cluster Headache: A Retrospective Cohort Study
Source: J Clin Med. 2026 Jul 30;15(15):5952. doi: 10.3390/jcm15155952 (PMC13467481; doi:10.3390/jcm15155952)
Supplement: Supplementary file 1 [file jcm-15-05952-s001.zip › Tables S1-S5.pdf]

Supplementary Table S1. STOP-Bang items and cutoffs for diagnostic performance for obstructive sleep apnea (Apnea Hypopnea index ≥ 5)

Cutoff	Sensitivity	Specificity	PPV	NPV	LR+	LR-
STOP-Bang ≥ 1	100.0 (91.4–100.0)	18.2 (5.1–47.7)	82.0 (69.2–90.2)	100.0 (34.2–100.0)	1.22	0.00
STOP-Bang ≥ 2	87.8 (74.5–94.7)	54.5 (28.0–78.7)	87.8 (74.5–94.7)	54.5 (28.0–78.7)	1.93	0.22
STOP-Bang ≥ 3	63.4 (48.1–76.4)	54.5 (28.0–78.7)	83.9 (67.4–92.9)	28.6 (13.8–50.0)	1.40	0.67
STOP-Bang ≥ 4	43.9 (29.9–59.0)	100.0 (74.1–100.0)	100.0 (82.4–100.0)	32.4 (19.1–49.2)	∞*	0.56
STOP-Bang ≥ 5	17.1 (8.5–31.3)	100.0 (74.1–100.0)	100.0 (64.6–100.0)	24.4 (14.2–38.7)	∞*	0.83
STOP-Bang ≥ 6	4.9 (1.3–16.1)	100.0 (74.1–100.0)	100.0 (34.2–100.0)	22.0 (12.8–35.2)	∞*	0.95
Snoring (S)	68.3 (53.0–80.4)	54.5 (28.0–78.7)	84.8 (69.1–93.3)	31.6 (15.4–54.0)	1.50	0.58
Tiredness (T)	65.9 (50.5–78.4)	45.5 (21.3–72.0)	81.8 (65.6–91.4)	26.3 (11.8–48.8)	1.21	0.75
Observed apnea (O)	46.3 (32.1–61.3)	100.0 (74.1–100.0)	100.0 (83.2–100.0)	33.3 (19.8–50.4)	∞*	0.54
High BP (P)	17.1 (8.5–31.3)	100.0 (74.1–100.0)	100.0 (64.6–100.0)	24.4 (14.2–38.7)	∞*	0.83
BMI ≥ 35 (B)	0.0 (0.0–8.6)	100.0 (74.1–100.0)	NA (NA–NA)	21.2 (12.2–34.0)	∞*	1.00
Age ≥ 50 (A)	17.1 (8.5–31.3)	100.0 (74.1–100.0)	100.0 (64.6–100.0)	24.4 (14.2–38.7)	∞*	0.83
Neck circ. (N)	7.3 (2.5–19.4)	100.0 (74.1–100.0)	100.0 (43.8–100.0)	22.4 (13.0–35.9)	∞*	0.93
Male (G)	95.1 (83.9–98.7)	27.3 (9.7–56.6)	83.0 (69.9–91.1)	60.0 (23.1–88.2)	1.31	0.18

Data is presented as average (95% CI). The 95% CIs were estimated using the Wilson method for binomial proportions. Bang = BMI (body mass index), age, neck circumference, and male gender; NPV = negative predictive value; PPV = positive predictive value; STOP = snoring, tiredness, observed apnea, and high BP (blood pressure), NA: Not available, due to the absence of subjects meeting the BMI ≥35 criterion.

* LR+ was undefined (∞) when specificity = 100%; LR− was undefined (∞) only when both sensitivity and specificity = 100%.

Supplementary Table S2. Incremental value of cluster-headache rhythmicity items over STOP-Bang for obstructive sleep apnea (Apnea Hypopnea index ≥ 10)

Candidate item	n (%)	Univariable OR (95% CI)	P-value*	STOP-Bang+item AUC	ΔAUC from STOP-Bang AUS	Adjusted OR (95% CI)	LRT p-value (incremental)	DeLong p-value	continuous NRI	NRI 95% CI	NRI P value
Circadian rhythmicity	35 (67.3)	1.06 (0.29–3.99)	1.000	0.757 (0.628–0.886)	-0.010	0.87 (0.23–3.31)	0.838	0.649	-0.373	-0.902 to 0.156	0.167
Nocturnal rhythmicity	17 (32.7)	1.33 (0.36–5.02)	0.769	0.780 (0.656–0.904)	+0.013	1.77 (0.45–6.94)	0.411	0.617	0.127	-0.383 to 0.638	0.625
Morning rhythmicity	9 (17.3)	0.48 (0.07–2.63)	0.469	0.756 (0.628–0.885)	-0.011	0.76 (0.15–3.98)	0.748	0.632	-0.279	-0.696 to 0.139	0.191
Seasonal rhythmicity	29 (55.8)	0.29 (0.08–1.02)	0.050	0.726 (0.591–0.861)	-0.041	0.48 (0.13–1.74)	0.265	0.115	-0.607	-1.123 to -0.091	0.021
Circadian and/or seasonal	45 (86.5)	0.13 (0.00–1.17)	0.046	0.734 (0.599–0.869)	-0.033	0.15 (0.01–1.66)	0.080	0.090	-1.064	-1.517 to -0.610	<0.001
CH onset age ≥ 30 (C30)	21 (40.4)	7.47 (1.91–34.69)	0.002	0.804 (0.688–0.919)	+0.036	5.58 (1.40–22.28)	0.011	0.125	0.910	0.433 to 1.387	<0.001

Each candidate item was added to the 8-item STOP-Bang score (N = 52 for all items). Nocturnal and morning rhythmicity are subtypes of circadian rhythmicity; patients without circadian rhythmicity were assigned absent for these subtypes.

* Univariable association of the item with OSA by Fisher's exact test (OR = odds of OSA, feature present vs absent).

Adjusted OR and LRT P from nested logistic regression (OSA ~ STOP-Bang vs OSA ~ STOP-Bang + item) the likelihood-ratio test is the formal test of incremental value for a nested addition.

DeLong P compares the STOP-Bang and STOP-Bang+item AUCs; continuous NRI also uses STOP-Bang as the reference and is reported as an exploratory measure only, not as primary evidence of incremental value (the formal test is the likelihood-ratio test). NE = not estimable (sparse/separated cell). C30 is the modification retained in the final model.

Supplementary Table S3. Internal validation of screening-score discrimination — bootstrap optimism correction (N = 52)

Screening tool	Outcome	Apparent AUC (95% CI)	Optimism	Optimism-corrected AUC
STOP-Bang (8-item score)	OSA (AHI ≥ 10)	0.767 (0.642–0.893)	0.000	0.767
STOP-Bang + C30 (unweighted score)	OSA (AHI ≥ 10)	0.804 (0.688–0.919)	0.000	0.804
STOP-Bang + C30 (re-weighted model)	OSA (AHI ≥ 10)	0.820 (0.707–0.933)	0.011	0.809
STOP-Bang + C30 (candidate re-selection)	OSA (AHI ≥ 10)	0.804 (0.688–0.919)	0.000	0.804
STOP-Bang (8-item score)	OSA (AHI ≥ 5)	0.767 (0.617–0.917)	0.001	0.766
STOP-Bang + C30 (unweighted score)	OSA (AHI ≥ 5)	0.782 (0.640–0.923)	0.001	0.781
STOP-Bang + C30 (re-weighted model)	OSA (AHI ≥ 5)	0.784 (0.637–0.931)	0.029	0.755

Optimism-corrected area under the ROC curve (AUC) from bootstrap resampling (Harrell's enhanced bootstrap; 2000 resamples for the fixed-score and re-weighted models, 1000 for the candidate re-selection model). In each resample the model was refit and its discrimination re-estimated on the resample and on the original cohort; optimism is the mean difference across resamples (floored at 0), and the optimism-corrected AUC equals the apparent AUC minus optimism. Optimism = 0.000 denotes a negative bootstrap estimate, i.e. no detectable overfitting.

STOP-Bang and STOP-Bang + C30 are pre-specified unweighted point scores entered as a single predictor; the near-zero optimism reflects the absence of data-estimated weights, so the apparent AUCs are not inflated by model fitting. The re-weighted model estimates separate coefficients for the STOP-Bang score and C30 (stricter check).

Candidate re-selection: because C30 was identified in this cohort by screening cluster-headache candidate items against STOP-Bang, the candidate selection (smallest nested likelihood-ratio-test P among circadian, nocturnal, morning, seasonal, circadian-and/or-seasonal, and C30) was repeated within each resample, so optimism from choosing C30 among candidates is also captured; C30 was re-selected in 58% of resamples for the primary outcome. C30 = cluster-headache onset age >= 30 years. Apparent AUC 95% CI by the DeLong method.

Supplementary Table S4. Modified screening models for obstructive sleep apnea (Apnea Hypopnea index ≥ 5)

Cutoff	Sensitivity	Specificity	PPV	NPV	LR+	LR-
Cluster onset age ≥ 30 (C30)	46.3 (32.1–61.3)	81.8 (52.3–94.9)	90.5 (71.1–97.3)	29.0 (16.1–46.6)	2.55	0.66
Circadian/Seasonal (R)	82.9 (68.7–91.5)	0.0 (0.0–25.9)	75.6 (61.3–85.8)	0.0 (0.0–35.4)	0.83	0.00
STOP+C30 (≥ 3)	51.2 (36.5–65.7)	90.9 (62.3–98.4)	95.5 (78.2–99.2)	33.3 (19.2–51.2)	5.63	0.54
STOP+R (≥ 3)	58.5 (43.4–72.2)	54.5 (28.0–78.7)	82.8 (65.5–92.4)	26.1 (12.5–46.5)	1.29	0.76
STOP-Bang+C30 (≥ 3)	73.2 (58.1–84.3)	54.5 (28.0–78.7)	85.7 (70.6–93.7)	35.3 (17.3–58.7)	1.61	0.49
STOP-Bang+C30 (≥ 4)	53.7 (38.7–67.9)	90.9 (62.3–98.4)	95.7 (79.0–99.2)	34.5 (19.9–52.7)	5.90	0.51
STOP-Bang+C30 (≥ 5)	24.4 (13.8–39.3)	100.0 (74.1–100.0)	100.0 (72.2–100.0)	26.2 (15.3–41.1)	∞*	0.76

B = BMI ≥ 27; C30 = onset age ≥ 30; R = rhythm; M = male.

* LR+ was undefined (∞) when specificity = 100%; LR− was undefined (∞) only when both sensitivity and specificity = 100%.

Supplementary Table S5. Polysomnographic and STOP-Bang findings by timing of polysomnography (cluster bout vs. remission)

Variable	Cluster bout (n=28)	Remission period (n=24)	P value
AHI (median, IQR)	7.8 (5.1–17.6)	10.6 (5.2–23.6)	0.693
STOP-Bang score (median, IQR)	3.0 (2–4)	3.0 (2–4)	0.620
OSA (AHI≥5), n (%)	22 (78.6%)	19 (79.2%)	1.000
Treatment-relevant OSA (AHI≥10), n (%)	12 (42.9%)	13 (54.2%)	0.578

P by Wilcoxon rank-sum (continuous) or Fisher's exact (categorical).
